# Cosmetic Procedures After Massive Weight Loss Surgery: A Guide for Prospective Patients

**DOI:** 10.7759/cureus.72864

**Published:** 2024-11-01

**Authors:** Marina Handal, Jenna Handal, Tiffany Nevill, Paige Finkelstein, Kandace Kichler

**Affiliations:** 1 Medicine, Dr. Kiran C. Patel College of Osteopathic Medicine, Nova Southeastern University, Fort Lauderdale, USA; 2 Biology, Nova Southeastern University, Fort Lauderdale, USA; 3 General Surgery, Franciscan Health Olympia Fields, Olympia Fields, USA; 4 General Surgery, Columbia University Business School, New York City, USA; 5 Cosmetic Surgery, Hass Plastic Surgery, Palm Beach, USA

**Keywords:** bariatric medicine, bariatric surgery severe obesity, body contouring after weight loss, cosmetic procedures, cosmetic surgeon, morbid obesity revisional bariatric surgery, weight reduction

## Abstract

In the United States, bariatric surgery has become the gold standard treatment for patients struggling with obesity and its severe sequelae. Often, after bariatric surgery, massive weight loss (MWL) occurs, leaving patients with cutaneous deformities such as excess tissue and redundant skin. As such, MWL patients struggle with cosmetic and functional impairments that hinder their quality of life, self-esteem, and body image. Here, we present the first guide to cosmetic surgical procedures available to patients following MWL to improve aesthetic and functional results after bariatric surgery. The guide features several body contouring procedures such as abdominoplasty, liposuction, body lift, brachioplasty, thighplasty, flankplasty, mastopexy, and facelift. This guide serves to educate patients about the surgical options they can elect after bariatric surgery in order to achieve their cosmetic and functional goals. Physicians or other medical staff may also use this guide as an aid for counseling MWL patients. Each surgical procedure is highlighted for the reader to understand its goals, expected outcomes, and patient-perceived cost-effectiveness. The guide seeks to be transparent about all benefits, risks, and options available to these MWL patients. Ultimately, the purpose of this guide is to inform post-MWL patients about their options for cosmetic procedures and to assist them in this critical health decision-making process.

## Introduction and background

As obesity rates in the United States continue to increase, the prevalence of patients opting for bariatric surgery has risen linearly. Following surgery, patients experience massive weight loss (MWL), which is defined as ≥50% loss of excess weight. Excess weight is determined by the BMI scale, which classifies the normal category as 18.5-24.9 kg/m^2^ and mild obesity as 27.0-29.9 kg/m^2^ [1]. The contour deformities that occur after rapid bariatric weight loss and the sudden shift in BMI lead to excess skin and soft tissue, as well as poor skin tone and muscle laxity. Typically, redundant tissue collapses in the lower abdomen, buttocks, and medial thigh, leading patients to experience pain and irritation under massive skin folds [1]. As such, many post-bariatric patients suffer from feelings of poor body image and diminished self-confidence despite their weight loss achievements. The literature supports that cosmetic procedures for MWL improve aesthetic satisfaction, body positivity, quality of life, and physical functioning for patients after bariatric surgery [2]. This guide aims to provide patients with existing evidence regarding the benefits, complications, and patient-perceived cost-effectiveness of various cosmetic procedures following bariatric surgery.

## Review

Abdominoplasty

Abdominoplasty is one of the most frequently performed cosmetic procedures indicated for post-operative bariatric patients following MWL. The overarching goals of these procedures are to remove excess skin and fat while tightening weakened muscles along the abdomen. Ideal aesthetic outcomes are characterized by a natural umbilicus and enhanced abdominal musculature/contour with the preservation of skin integrity. Liposuction is typically used to attain a more defined waistline [3]. It is particularly elected by the surgeon if the curvature of the abdomen at the waist is blunted or cuboid [4]. Markings made for the liposuction include a straight line extending from the superior iliac spine to a flank roll with the skin under tension in order to mimic post-operative tension; this procedure is performed by the surgeon in order to minimize the risk of a high-riding scar [3]. Furthermore, mesh reinforcement or rectus sheath plication may be used by the surgeon to strengthen the abdominal wall. Rectus sheath plication aims to efface skin laxity, simultaneously ensuring limited tension of the skin [4]. These surgical options are paramount in helping patients achieve both cosmetic and functional therapeutic benefits. Subsequently, the ideal candidates for full abdominoplasties are patients suffering from excessive skin laxity, fat, and abdominal wall weakness [5].

Associated complications of abdominoplasties include seromas and hematomas, which occur in around a third of cases. If untreated, these common post-operative risks can result in necrosis or infection of the surgical incisions. Complications can be minimized with closed-suction drainage systems, thereby lessening fluid accumulation. It is also critical that the surgeon preserves the vascular supply to the umbilicus after abdominoplasty. Superficial wounds are the most common complication of abdominoplasties, followed by infection and wound dehiscence due to excessive tension on the sutures [3]. Wound healing depends on proper nutritional status and identifying preliminary risk factors of the individual patient prior to surgery. Compared to the postpartum patient, an MWL patient is more likely to experience back pain, intertrigo, and nutritional deficiencies. Despite significant weight loss, an MWL patient tends to have a higher BMI, which translates to a greater risk of surgical complications such as soft tissue deformity. Additionally, aesthetic issues with pre-existing scars along the abdominal wall must be considered when performing an abdominoplasty on an MWL patient [6].

Patients undergoing an abdominoplasty had a 98% satisfaction rate and were found to experience an improved quality of life, including a higher self-esteem [4]. Around 88.8% of patients who underwent liposuction and an abdominoplasty, either together or alone, had high levels of satisfaction. It is important to note that the discomfort level of undergoing an abdominoplasty alone is similar to that of the combined liposuction-abdominoplasty procedure. The combined procedure generated the highest patient satisfaction of 99.2% [5]. According to the Aesthetic Plastic Surgery National Databank, the average cost for an abdominoplasty is $6,173 versus liposuction at $3,382 [7].

Liposuction

It is critical to highlight the importance of liposuction and its use in cosmetic surgeries for MWL patients. Liposuction, or suction-assisted lipectomy, is a procedure that removes adipose tissue from the subcutaneous space, with the goal of enhancing the body’s contour. Liposuction is most commonly used for fat removal between the inframammary and gluteal fold, and it is often performed with abdominoplasty, breast augmentation, gluteal fat transfer, and other aesthetic procedures. Liposuction is also used during reconstructive surgery for the breast and face. Outside of bariatrics, it is the second most commonly performed surgery in the United States. In fact, it is the most elected surgical operation for patients between 35 and 64 years of age [8]. In MWL patients, liposuction is critical in providing patients with a more desirable body contour but typically cannot be used as the sole modality to achieve optimal aesthetic outcomes. MWL patients often have inflated thighs, and thus the surgeon may elect circumferential liposuction and then perform an excision after six months. Patients may find that the contour of the thighs worsens prior to this excision [9]. The roles and staging of liposuction must be tailored to each MWL patient. Simultaneous liposuction and rejuvenation allow for an area to be treated without extra surgery. However, performing both procedures at once substantially increases the risk of edema and, thus, vascular compromise to the flaps. Another option is to perform liposuction six months prior to excision, which allows for debulking of the area and, therefore, minimizing the rate of complications. Liposuction alone cannot provide the desired aesthetic results in the post-operative bariatric patient population [1].

The average national cost of liposuction is $3,300 [7]. The use of liposuction in body contouring among post-bariatric patients displayed a 67% satisfaction rate in a large cohort of patients, as demonstrated in a study published by The European Journal of Obesity. The study also describes that weight status, as expressed by the percentage of excess weight shed prior to contouring, directly correlates to the risk of developing post-operative complications. As such, BMI remains a significant risk factor for complications following liposuction. Seromas, infections, and hematomas are other notable complications seen in this patient population following the procedure [10].

Body lift

Circumferential body lifts are becoming the method of choice in cosmetic surgery for post-operative bariatric patients following MWL. This is because MWL patients are unique in that they suffer from atonic skin quality with increased laxity and generally require tissue resection [11]. A body lift entails a buttock and abdominoplasty, and it is often simultaneously performed with a thighplasty. This compounded procedure addresses both the functional and aesthetic concerns of patients. As such, the procedure allows for the reconstruction and restoration of the aforementioned areas by improving the volume and shape of the afflicted regions [12].

The primary goals of a total body lift are tailored according to each region. In the abdominal region, the surgeon aims to achieve maximal skin and fat reduction with muscle contouring in the abdominal wall. The waist appears contoured and trimmed through major excision of the subcutaneous abdominal fat. Moreover, the flanks are restored through vertical tissue resection, with fleur-de-lis procedures used in a manner of horizontal tissue resection for trimming of the waistline. Lastly, the treatment approach for the gluteal region depends on the patient’s preoperative status and must be assessed by the surgeon on a case-by-case basis; all options set out to improve gluteal volume and shape [11]. The Aesthetic Plastic Surgery National Databank reports that the procedure cost can range from $7,000 to $30,000, depending on whether a lower, upper, or total body lift is performed [7].

One of the most common risks of the procedure is the development of pulmonary embolism and deep vein thrombosis, which can be managed effectively with appropriate prophylaxis. Such complications are especially concerning among smokers and patients taking oral contraceptives. Patients are informed of this risk and advised to discontinue hormonal therapies four weeks before surgery. Other risks associated with circumferential body lifts include paresthesia and persisting numbness, with seromas typically afflicting the abdominal region [11]. The Aesthetic Surgery Journal found that MWL patients most often experienced complications relating to wound healing. These patients will also likely face complications such as thrombotic rate and seromas. The studies reported that total body lifts are associated with a massive reduction in observable deformities in post-operative bariatric patients and a high patient satisfaction rate in aesthetic and functional outcomes. Total body lift surgery is effective as it combines multiple body contouring operations into one single-stage surgery; this comprehensive approach to cosmetic surgery supports a continuum of body contouring. Compared with a total body lift, multiple operations may pose increased risks of infection and thrombophlebitis, as well as an increased need for blood transfusions [13].

Brachioplasty

Another cosmetic operation that MWL patients opt for is brachioplasty. This procedure provides contour improvement by correcting severe arm deformity. Such deformity entails excess, loose skin in the posterior axillary fold and its extension down the arm. This is often compounded by lateral chest rolls of skin and flattening and lengthening of the anterior axillary fold. Liposuction of the arm can also be combined with brachioplasty to optimize skin tone, contour, and proportions [14]. There are several presentations that need to be considered. For instance, many patients present with over-inflated arms even after weight loss. It is recommended that initial liposuction takes place to deflate the arms before proceeding to surgical excision six months later. Traditional brachioplasty procedures use a medially placed scar, which typically extends from the distal arm to the dome of the axilla. It is cited that this technique and scar placement lead to the most aesthetically favorable outcomes for MWL patients by minimizing the visibility of post-surgical distortions [15].

The associated risks after brachioplasty include superficial infections, seromas and hematomas, sensory nerve injury, recurrent skin laxity, and asymmetry. These complications are reported to occur as much as 56%. The most common complications with brachioplasty were identified as infection and hematoma [15]. Zomerlei et al. found that hypertrophic scarring occurred as frequently as 24% of all cases under study, in turn necessitating an 8.3% surgical revision to obtain optimal results in MWL patients. The second most common major complication was infection, with a rate of 14.6%. Because MWL patients often bear thin skin in the upper arm region, they are more prone to open wounds and scarring. Tension-free closures, eversion of the wound edge, and using occlusive dressings such as taping and glue allow surgeons to circumvent unnecessary complications [16]. The American Society of Plastic Surgeons cites the average national cost for a brachioplasty as $4,680 [7].

The surgeon must take on extensive perioperative care when performing a brachioplasty. Ideally, the best candidates for surgery are those whose weight has remained stable for more than six months. In the preoperative stages, it is critical to ensure that the incision line is not extended too distally, past the medial epicondyle, as damage commonly occurs to the medial antebrachial cutaneous nerve. Thus, the scar length is adjusted according to arm deformity. Scarring at the joint may also lead to restriction of the range of motion at the elbow [17].

Thighplasty

The thigh lift, or “thighplasty,” is a procedure that effectively reduces skin laxity of the thigh and removes excess skin. It is commonly combined with liposuction to decrease fat volume as well [18]. The extent of the thighplasty depends on the amount of skin laxity; for example, a patient with laxity located at the upper third of the thigh would only be subjected to a horizontal excision [19]. The surgery requires a lower body lift and is followed by the excision of excess skin. The excision of medial thigh skin, use of liposuction, and integration of prone and supine operative positions are essential to thigh contouring. Thighplasty improves thigh contour and reduces lower torso and thigh laxity with unobtrusive scarring. MWL patients tend to have loose skin on the upper posterior thigh, sag and rolls of skin on the medial thigh, and stacked skin on the anterior thigh. These patients also have loose skin on their upper lateral thigh that stops at the midthigh, forming saddlebags. Thighplasty corrects these deformities and makes the skin taut [20].

Although aesthetic reshaping is achieved, several complications are associated with this procedure. There is an increased chance of risk due to the skin’s thinness and weakness. The post-bariatric patients who opt for this cosmetic surgery have atrophic dermis susceptible to wound dehiscence as well as scar enlargement and migration. Because the inner thigh is a more humid area, wounds tend to heal at a slower pace. These wounds are an increased risk of infection due to their proximity to the anus and genitals. Deformation of the labia majora and vagina may be caused by scar retraction in the inguinal area [21]. One of the more common complications after thighplasty is seroma formation, which results from failing closed-suction drains, compression garments, and sclerosing agents. Patients are also at high risk of lymphedema, swelling caused by the injury of lymphatic channels, and suture granulomas [22]. It is important to note that, according to a 2016 Aesthetic Surgery Journal study, thighplasty patients had higher rates of complications within hospitals than in ambulatory settings. The studies found that the most common major complications associated with thighplasty are infection, hematoma, fluid overload, and venous thromboembolism [23]. Nonetheless, a 2018 study conducted by the Journal of Plastic, Reconstructive & Aesthetic Surgery revealed that post-operative patient satisfaction was high: 72% of thighplasty patients were completely satisfied with their results, while 8% were partially satisfied [24]. As noted by the Aesthetic Plastic Surgery National Databank in 2019, a thighplasty procedure costs an average of $5,030 [7].

Flankplasty

MWL patients tend to also opt for a flankplasty procedure, which usually involves liposuction. Flankplasty incorporates procedures for both the lower and upper body. The lower body contouring includes flattening of the abdomen, excision of lower back rolls, and relocation of the navel. The waistline and buttocks are redefined, the mons pubis are reduced, and the anterolateral thigh is elevated. In contrast, upper body surgery is focused on the excision of mid and upper back folds and the reshaping of arms by excision of loose skin and redundant rolls. The inframammary fold is also recreated [25].

After bariatric surgery, the patients tend to have excess adiposity and soft-tissue laxity of the back. Typically, patients have laxity extending from the neck to the lower back [25]. The excess skin on the back can migrate inferiorly, and flank deformities can develop [26]. Rolls of the lower abdomen and pubic area create an overhanging pannus and cause pain and irritation [1]. Flankplasty is beneficial in that it improves soft tissue contour from the upper back to the lumbar area. Soft tissue folds of the middle and upper back are also eliminated [25]. The removal of these redundant folds can improve a patient’s physical function and quality of life [27,28]. Both the aesthetic and functional aspects of a patient’s life are favored [28]. Notably, flankplasty coupled with lipoabdominoplasty was found to correct flank deformities, resulting in a deepening of the waist and a tighter lower torso [26].

The flankplasty procedure can result in several complications. One of the main post-operative issues is wound dehiscence. Peri-operative tobacco use is noted as a plausible risk factor for complications following lower body contour [27]. Wound dehiscence is most seen in the lower back, possibly leading to the development of seroma. Other potential risks are hematoma, lymphoedema, and the improper appearance or loss of the navel [1]. It is important to note that a patient with a BMI greater than 30 kg/m^2^ may have an increased complication rate [27]. According to the Journal of Plastic, Reconstructive & Aesthetic Surgery, patients were shown to have a 98% satisfaction rate ranging from pleasing to excellent. The Aesthetic Plastic Surgery National Databank revealed in 2019 that this surgery costs an average of $7,768 [7].

Mastopexy

After bariatric surgery, the breasts tend to have severe ptosis and asymmetry, as well as a reduction in lateral curvature and upper pole volume. The areolas may not be centered, the inframammary folds may be loose, and lateral chest wall rolls may be present. The mastopexy procedure helps MWL patients with the restoration and reshaping of the breasts [29]. It removes excess skin, lifts the nipple-areola complex, and medializes the breasts, improving the patient’s upper body contour. Ultimately, the fullness and symmetry of the breasts are restored, and the ptosis is corrected. Breast volume can be restored by using excess tissue in the epigastric region or lateral thoracic region found in patients following MWL [30].

MWL patients have poor inframammary support and a lack of skin elasticity [29]. Some risks of performing a mastopexy procedure on an MWL patient include ptosis relapse, reduction of upper pole volume, and upward migration of the nipple-areola complex. Other complications contributing to less patient satisfaction include scar location and extension [30]. Post-operative ptosis was found to be significantly related to age. Another complication that MWL patients may face is implant malposition following surgery, and it is significantly correlated to a higher BMI [29]. The patient has the potential to suffer from thromboembolic events as well as seromas and hematomas. Thromboembolic events can be prevented with special measures directed by the surgeon. For example, elastic-compression garments and low molecular weight heparin may be effective in preventing a thromboembolic event. The patient may face recurrent ptosis, as well as nipple necrosis or nipple loss. The patient’s risk of nipple necrosis or loss is dependent on their relationship with smoking, alcohol, or drugs. With regard to aesthetics, the patient may have unfavorable, visible scarring [31]. Despite these complications, a 2019 study conducted by the Journal of Cutaneous and Aesthetic Surgery showed that 100% of mastopexy patients were somewhat to extremely satisfied with their results. As indicated by the Aesthetic Plastic Surgery National Databank in 2021, a mastopexy procedure costs an average of $4,864 [7].

Facelift

MWL patients also have significant deflation of their central face’s soft tissue envelope; this deflation is shown by excess skin at the central neck, deepened nasolabial folds, and malar descent. In conjunction, MWL patients tend to have persistent skin laxity on their face and neck. There may be less of an ability to contract the skin due to soft tissue deflation. These post-operative outcomes lead the patient to opt for a facelift. A facelift allows an MWL patient the opportunity to replenish their face’s volume and remove unnecessary, excess skin. The procedure may also include rejuvenation of the neck in order to give the patient a more complete lift. This particular procedure is known to significantly reduce an individual’s apparent age and improve their cervicofacial appearance [32]. A facelift eliminates the droopiness and deflation of the face and neck, ultimately defining the jawline and facial contouring. Surgeons often combine this cosmetic surgery with fat compartment augmentation. This procedure is particularly needed for MWL patients with deflated facial fat in the midface [33]. It was noted that skin excision and tightening, without the incorporation of fat transfer, may generate results that are less than ideal [32].

Post-bariatric surgery, a patient who does a facelift may be at risk of soft tissue deflation, hematomas, and seromas [32]. It is essential to highlight that a 2016 study conducted by the Aesthetic Surgery Journal found a correlation between a facelift patient’s smoking habit and the development of a hematoma [7]. An MWL patient may be more likely to develop a hematoma since they must undergo a greater degree of excess skin removal and thus experience more tissue loss. Hypertrophic scarring is another complication that may arise. It is especially critical for the MWL patient that the surgeon thoroughly plans skin incisions and anticipates repositioning of skin flaps. Compared to a non-MWL patient, an MWL patient has larger amounts of excess skin at the lower neck, requiring longer incisions at the posterior hairline. In addition, it is expected that MWL patients will more often experience persistent skin laxity following the facelift procedure. Therefore, there may be a need for a revision facelift in order to receive optimal results [32]. In 2010, the Plastic and Reconstructive Surgery Journal found high patient satisfaction in the short-term and long-term follow-up. 97.8% of patients were satisfied with their facial appearance a year after their facelift procedures [34]. The Aesthetic Plastic Surgery National Databank stated that a facelift in 2021 costs an average of $9,127 [7].

## Conclusions

For patients who may not yet have consulted with a physician or would like data to review, this is the first comprehensive guide available to MWL patients who seek to learn more about the various ways in which cosmetic surgery can assist them in achieving their desired aesthetic and functional outcomes. Through the information provided, we hope to assist MWL patients in establishing their goals and expectations of cosmetic surgery. Before any surgery, psychological and nutritional consultations are critical in managing patient expectations for post-operative aesthetic outcomes following MWL. Thus, it is critical to educate patients on the need for major lifestyle changes following the procedure, including daily physical exercise, a balanced diet, and the elimination of smoking so that the best outcomes can be achieved for the patient.
